# Agreement Between a Wireless Sleeve and a Goniometer in Measuring Elbow and Knee Range of Motion

**DOI:** 10.7759/cureus.91748

**Published:** 2025-09-06

**Authors:** Eric V Neufeld, Simran R Desai, Brandon J Klein, Michael J Meade, Lucas E Bartlett, Randy M Cohn, Nicholas A Sgaglione

**Affiliations:** 1 Orthopedic Surgery, Northwell Health, New Hyde Park, USA; 2 Orthopedic Surgery, Long Island Jewish Medical Center, New Hyde Park, USA; 3 Orthopedic Surgery, Long Island Jewish Valley Stream, Valley Stream, USA; 4 Orthopedic Surgery, Donald and Barbara Zucker School of Medicine at Hofstra/Northwell, Hempstead, USA

**Keywords:** goniometer, orthopedic sensor technology, orthopedic sleeve, range of motion, wearable technology

## Abstract

Introduction

Goniometers are a common clinical tool for measuring joint range of motion. Wearable sleeves offer a novel and alternative method to measure joint angles, record range of motion (ROM), and remotely track changes in ROM over time. Additionally, they may aid in the surgical planning required for deformity correction. This investigation compared the reliability and validity of a conventional goniometer to a novel transducer-embedded sleeve when measuring flexion and extension of the elbow and knee.

Methods

This was a cross-sectional study of 55 patients (110 elbows, 110 knees) who donned an elbow and knee sleeve embedded with a gallium-based strain sensor. Flexion and extension of the elbow and knee were measured both manually using a goniometer and a wearable sleeve. Agreement between the two devices was assessed via Bland-Altman plots.

Results

Regarding elbow flexion, the bias observed between the two devices was 4.6° with LoA spanning from -14.9° to 24.1°. Regarding elbow extension, the bias observed between the two devices was -9.8° with LoA spanning from -25.6° to 6.0°. Regarding knee flexion, the bias observed between the two devices was -4.6° with LoA spanning from -35.6° to 26.4°. Regarding knee extension, the bias observed between the two devices was -2.8° with LoA spanning from -8.9° to 3.3°.

Conclusion

Wearable sleeves show promise for clinical ROM measurement and remote monitoring but require further refinement to reduce bias before clinical application.

## Introduction

Goniometers are the traditional and practical clinical tool for measuring joint range of motion (ROM). Techniques have been described for measuring flexion and extension of the knee as well as flexion, extension, pronation, and supination of the elbow. However, despite its regard as the gold standard, the conventional goniometer is error-prone [1], time-consuming, and has a limited capacity to accurately measure more complex joint motions [2].

Wearable technologies demonstrate tremendous promise in advancing orthopedic care through physical examination and assessment [3]. Specifically, biotechnological wearable sleeves offer an alternative method to measure joint angles, record ROM, and remotely track changes in ROM over time [4]. Furthermore, they may aid in the surgical planning required for deformity correction [4]. These sleeves employ a combination of textiles and malleable electronics, such as gallium-based alloys, which are deemed useful in both wearable and implantable devices, as well as soft robotics [5]. They have been theorized to help coordinate treatment plans [4], facilitate corrective action (i.e., implementing exercises and bracing to correct alignment issues and halt progression of malalignment) [4], and monitor recovery and performance metrics in post-injury and post-operative patients [4]. In addition to deformities, orthopedic sleeves may also assist in the diagnosis of fractures and ligamentous injuries [4]. Furthermore, these measurement techniques can be used to fine-tune prosthetics and orthotics to accommodate specific joint angles and desired ROM to accommodate patients' anatomical differences while optimizing functionality [4].

While the potential clinical applicability of wearable sleeves is evident, a paucity of literature exists that validates these devices for routine clinical use. Therefore, the purpose of this investigation was to compare the agreement between a conventional goniometer and a novel sleeve in measuring flexion and extension of the elbow and knee.

## Materials and methods

Participants

Fifty-five participants, comprised of 27 males (49.1%) and 28 females (50.9%), with a mean age of 33.6±8.8 years and a total of 110 elbows and 110 knees, were evaluated. The study cohort consisted of healthy employee volunteers from two different hospitals within the same health system, who were recruited through word-of-mouth. Exclusion criteria included individuals with elbow or knee contractures or hyperlaxity. This included participants who could not achieve 90° of flexion or had at least a 5° loss of terminal extension. This investigation was approved by the Institutional Review Board at the Feinstein Institutes for Medical Research of Northwell Health under identification #23-0428. All participants provided written informed consent.

Study device

**Figure 1:**
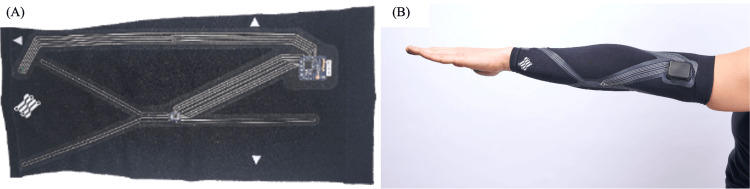
MetalGel sensors and arm sleeve. (A) MetalGel strain sensors (Portland, OR: Liquid Wire), interconnects, and circuit board on fabric. (B) Fully assembled arm sleeve.

**Figure 2:**
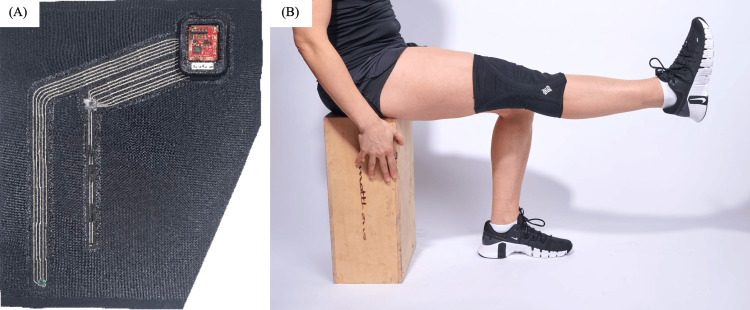
MetalGel sensors and knee sleeve. (A) MetalGel strain sensor (Portland, OR: Liquid Wire), interconnects, and circuit board on fabric. (B) Fully assembled knee sleeve.

Measurements were made using wearable sleeves (Portland, OR: Liquid Wire) containing proprietary gallium-based strain sensors (Figures 1A, 1B, 2A, 2B). The strain sensors were heat-bonded to the fabric of the sleeves and were aligned within the sleeves to provide the greatest amount of signal change during the desired joint movements. Signals from the sensors were transported to an analog-to-digital board via proprietary gallium-based interconnects and streamed via Bluetooth to the data collection computer. Data were sampled at 200Hz.

Study design and data collection

**Figure 3:**
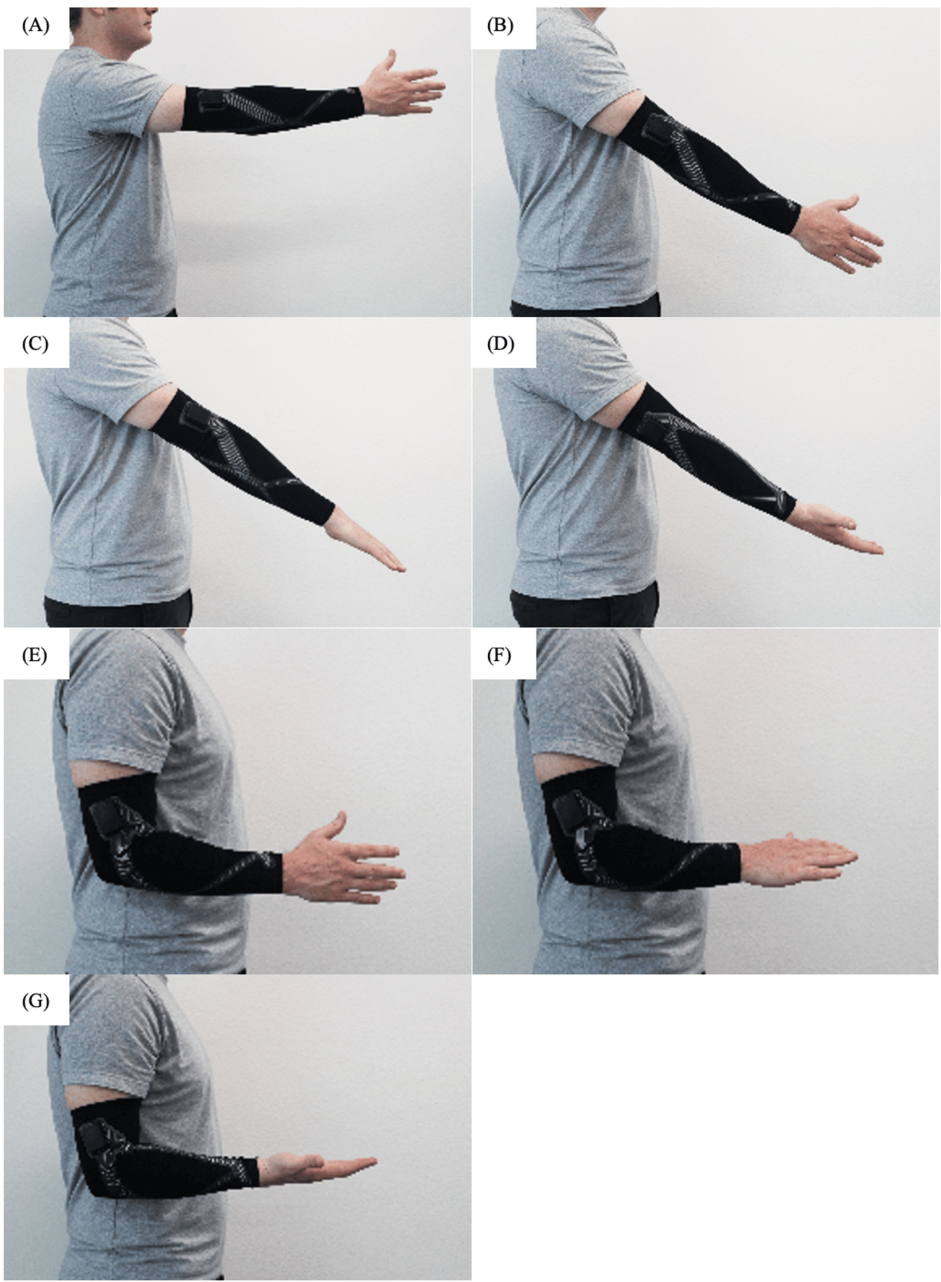
Static positions used to calibrate the arm sleeve. (A) 90° forward flexion at the shoulder, (B) 45° shoulder flexion, (C) forearm pronated at 45° shoulder flexion, (D) forearm supinated at 45° shoulder flexion, (E) shoulder adducted to 0° with elbow flexed to 90° and forearm neutral, (F) shoulder adducted to 0° with elbow flexed to 90° and forearm pronated, and (G) shoulder adducted to 0° with elbow flexed to 90° and forearm supinated.

This was a cross-sectional study conducted over two months from September to November 2024. For elbow measurements, participants were fitted with the arm sleeve of an appropriate size, ranging from small to extra large, and began a conditioning period by cycling through full extension (forearm pronated) to maximal flexion (forearm supinated) three times. Calibration was then performed by holding the upper extremity in a series of positions for at least one second (Figures 3A-3G).

The first position consisted of 90° of forward flexion at the shoulder with the elbow fully extended and the forearm in neutral rotation. Next, the arm was lowered to 45° of shoulder flexion. The forearm was then held in pronation, followed by supination. Subsequently, the upper arm was adducted to 0° (i.e., held against the side of the trunk) with the elbow flexed to 90° and forearm held in neutral. The forearm was then held in 90° of pronation, followed by 90° of supination.

After calibration was complete, measurements were obtained. The elbow was first held in full extension (forearm pronated) and then in maximal flexion (forearm supinated). The circuitry in the sleeve calculated the degrees at these two positions and then wirelessly transmitted the data to proprietary software. Angles at these two positions were then measured manually via a goniometer and recorded by two examiners. This process was repeated with the arm adducted to 0° and the elbow flexed to 90°, first in maximal pronation and then in maximal supination. All measurements were performed bilaterally and repeated three times.

**Figure 4:**
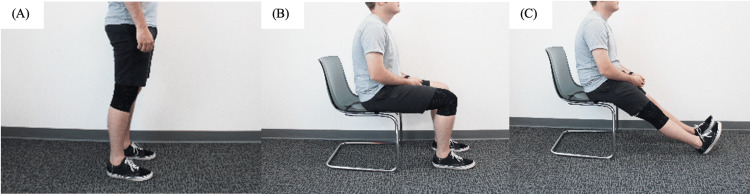
Static positions used to calibrate the knee sleeve. (A) Neutral stance with feet shoulder-width apart, (B) sitting on a chair with both knees flexed to 90°, and (C) sitting on a chair with both knees fully extended, forming an approximate 45° angle between the lower extremities and the floor.

After completing the elbow measurements, subjects were then fitted with a knee sleeve of the appropriate size, ranging from small to extra large. The examiners were trained on how to don the sleeves appropriately. A conditioning period began by cycling the knee while standing from full extension to maximal flexion three times. Calibration was then performed by holding the lower extremity in a series of positions for at least one second (Figures 4A-4C).

First, the participant stood in a neutral stance (with straight knees) and feet shoulder-width apart. Next, the participant sat in a chair with both knees flexed to a 90° angle. Then, from this position, both knees were fully extended, forming an approximate 45° angle between the lower extremities and the floor. After calibration was complete, measurements were obtained. The participant lay down on the ground with the target knee in full extension; they then maximally flexed it with the plantar surface of the foot flat on the floor. The knee joint angle at full extension and maximal flexion was recorded first by the sleeve and then manually via the same goniometer by the same two examiners using standardized workflows, with three measurements taken per subject.

Data analysis

Data were recorded as mean (standard deviation). The agreement between the ROM measured by the sleeves and the goniometer was assessed via bias and limits of agreement (LoA) derived from Bland-Altman plots [6]. Given the number of elbows and knees in each group, the central limit theorem was employed to assume each dataset approached a Gaussian distribution. Systematic bias was defined as present if the 95% confidence interval of each group’s bias did not include zero, and LoA, by convention, were set to ±1.96 standard deviations [7]. No p-values are utilized in this form of analysis. Using an a priori power analysis from a previous study of similar methodology [8], a sample size of 16-58 participants (or 32-116 individual joints) was calculated. All statistical analyses were performed in R version 4.4.1 (Vienna, Austria: R Foundation for Statistical Computing).

## Results

A total of 55 participants (110 elbows and 110 knees) met the inclusion criteria and were recruited for the study. After excluding data processing errors and significant outliers, 98 (flexion) and 96 (extension) elbows as well as 104 (flexion) and 106 (extension) knees were included for analysis. Mean elbow flexion was 130.7° as calculated by the sleeve compared to 135.3° by the goniometer, while elbow extension was 10.3° and 0.5° as measured by the sleeve and goniometer, respectively. Mean knee flexion was 135.1° as calculated by the sleeve compared to 130.6° by the goniometer, while mean knee extension was 3.1° and 0.3° as measured by the sleeve and goniometer, respectively.

**Figure 5:**
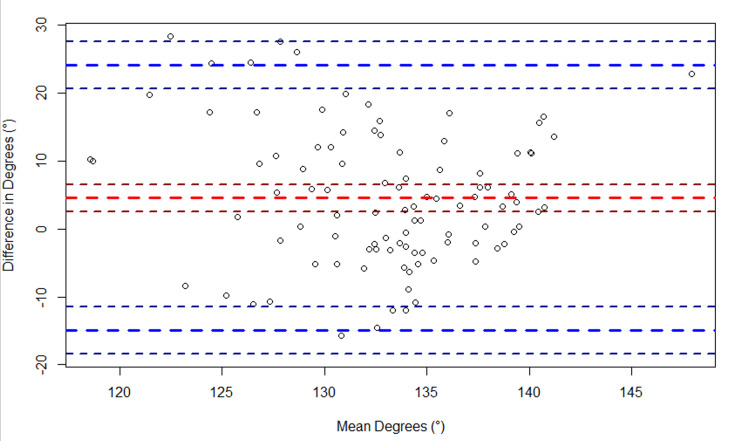
Bland-Altman plot of elbow flexion. The plot shows the difference in elbow flexion (goniometer - sleeve) versus the average of the two measurements ({goniometer + sleeve}/2). The large dashed red line indicates the bias, and the large dashed blue lines indicate the upper and lower limits of agreement (LoA). The smaller dashed lines bordering each large dashed line represent the 95% confidence intervals.

**Figure 6:**
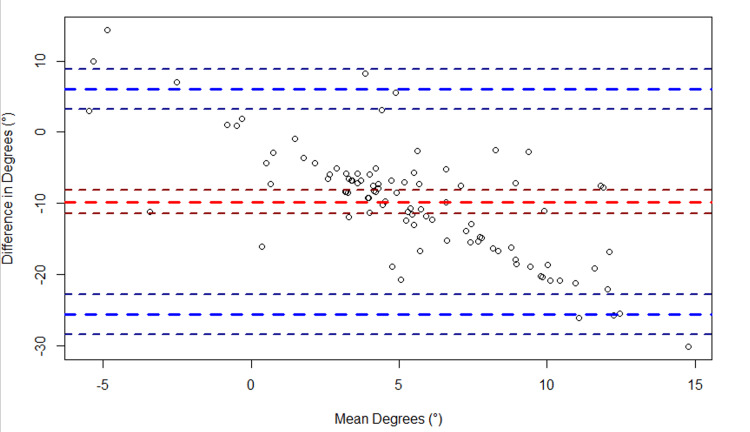
Bland-Altman plot of elbow extension. The plot shows the difference in elbow extension (goniometer - sleeve) versus the average of the two measurements ({goniometer + sleeve}/2). The large dashed red line indicates the bias, and the large dashed blue lines indicate the upper and lower limits of agreement (LoA). The smaller dashed lines bordering each large dashed line represent the 95% confidence intervals.

**Figure 7:**
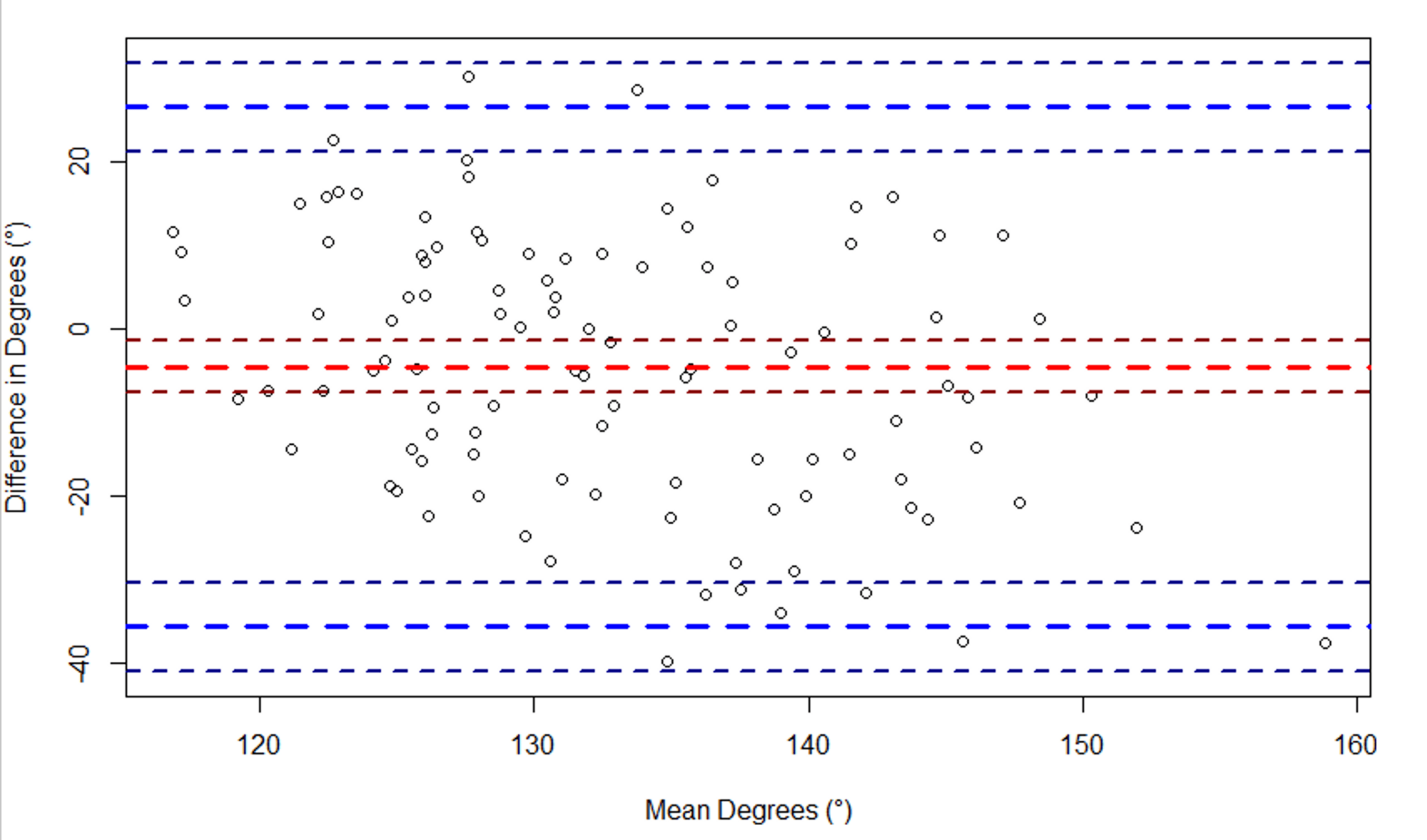
Bland-Altman plot of knee flexion. The plot shows the difference in knee flexion (goniometer - sleeve) versus the average of the two measurements ({goniometer + sleeve}/2). The large dashed red line indicates the bias, and the large dashed blue lines indicate the upper and lower limits of agreement (LoA). The smaller dashed lines bordering each large dashed line represent the 95% confidence intervals.

**Figure 8:**
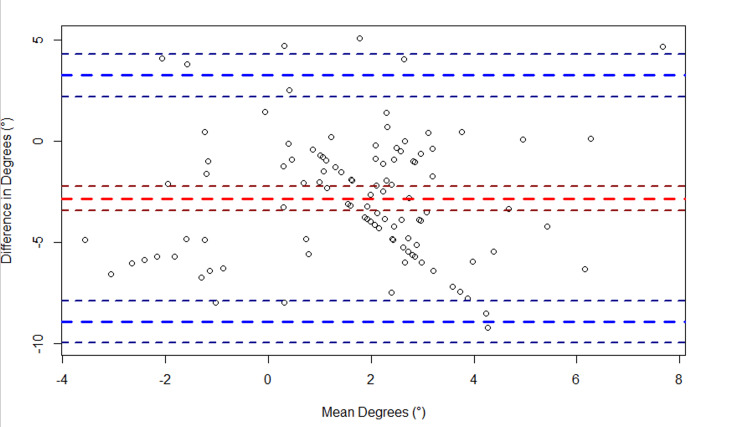
Bland-Altman plot of knee extension. The plot shows the difference in knee extension (goniometer - sleeve) versus the average of the two measurements ({goniometer + sleeve}/2). The large dashed red line indicates the bias, and the large dashed blue lines indicate the upper and lower limits of agreement (LoA). The smaller dashed lines bordering each large dashed line represent the 95% confidence intervals.

Regarding elbow flexion, a bias of 4.6° (95% CI: 2.6°, 6.6°) was observed between the two devices (Figure 5). The LoA spanned from -14.9° (95% CI: -18.4°, -11.5°) to 24.1° (95% CI: 20.7°, 27.6°). Regarding elbow extension, a bias of -9.8° (95% CI:- 11.4°, -8.2°) was observed between the two devices (Figure 6). The LoA spanned from -25.6° (95% CI: -28.5°, -22.8°) to 6.0° (95% CI: 3.2°, 8.9°). Regarding knee flexion, a bias of -4.6° (95% CI: -7.6°, -1.5°) was observed between the two devices (Figure 7). The LoA spanned from -35.6° (95% CI: -40.9°, -30.2°) to 26.4° (95% CI: 21.1°, 31.8°). Regarding knee extension, a bias of -2.8° (95% CI: -3.4°, -2.2°) was observed between the two devices (Figure 8). The LoA spanned from -8.9° (95% CI: -9.9°, -7.9°) to 3.3° (95% CI: 2.2°, 4.3°).

## Discussion

Systematic bias (i.e., the 95% CI for bias did not include zero) was found between the sleeve and goniometer in all four ROM measurements, indicating that the sleeve may require further refinement prior to clinical application. The sleeve underestimated elbow flexion but overestimated knee flexion, knee extension, and elbow extension. Measurements of knee extension demonstrated the smallest bias, where the sleeve overestimated the goniometer by 2.8°. Similarly, knee extension also yielded the narrowest LoA, spanning 12.2°. In contrast, elbow extension exhibited the largest bias, where the sleeve overestimated the goniometer by 9.8°. However, knee flexion demonstrated the widest LoA, spanning 62.0°. Considering that the participants in this investigation were primarily healthy adults without any known joint contractures or any other elbow or knee pathology, it is logical that knee extension exhibited the smallest bias and narrowest LoA, as nearly all individuals were able to achieve full extension as measured by the goniometer. From an engineering perspective, the sleeve was likely more accurate in measuring a joint that simulated a straight line rather than having to account for the angle generated by flexion. However, this explanation is not definitive, as most participants were also able to achieve complete elbow extension, yet this measurement yielded the largest bias. This discrepancy may be related to the ease of sleeve wear. The correct placement of the knee sleeve was guided by a circle that aligned with the patella, facilitating ease and verification of proper placement. On the contrary, donning the elbow sleeve required more precision, as a series of lines had to overlay the olecranon in the correct orientation; therefore, it is possible that some of the measurement error may have stemmed from small deviations in the wear of the elbow sleeve.

Smartphone applications comprise another form of technology utilized to measure ROM in place of a goniometer. A systematic review of 37 studies reported adequate relative agreement (e.g., inter-rater reliability/intraclass correlation coefficient) but inferior absolute agreement (e.g., mean difference±LoA) between the mobile applications and the criterion instrument (most commonly a goniometer) [9]. These authors concluded that an LoA range greater than ±9.8° was “poor” and less than ±9.8° was “good.” In the context of the present investigation, only measurements of knee extension would fall into the “good” category. This suggests that the sleeve requires further refinement before it can be used in routine clinical settings. However, it is important to note that the review incorporated studies focused on other joints, such as the spine and shoulder, rather than solely the elbow and knee. Therefore, their criterion parameters may not be fully applicable to the present data.

A different investigation that employed a 3D-printed wearable goniometer for the knee with a mobile application interface demonstrated more precise results [10]. At 0°, 30°, 60°, and 90° of knee flexion, the wearable device calculated a flexion angle with a precision of 1° relative to a goniometer. This comparison, however, is difficult to extrapolate to the agreement calculations in the present study. Another investigation compared the reliability of an inclinometer, smartphone application, and goniometer in a cohort of 92 patients with an anterior cruciate ligament injury or status post reconstruction [11]. These authors reported that the goniometer exhibited the lowest inter-rater reliability and recommended using the inclinometer over both the goniometer and smartphone application.

Other wearable sensors have been utilized to assess elbow biomechanics in throwing athletes. One study of 10 healthy baseball pitchers with either collegiate or professional experience compared various shoulder and elbow kinematics between a wearable elbow inertial measurement unit (IMU) and a criterion motion capture system [12]. The authors reported a moderate to strong correlation for elbow varus torque but a poor correlation for elbow extension speed. However, the authors concluded that because of the high reproducibility of the elbow IMU’s data, the device may be acceptable for casual use, especially to monitor changes in the same athlete over time. A similar study of the same elbow IMU in 10 healthy high-school varsity baseball pitchers concluded that the device underestimated elbow varus torque by 20-60% but demonstrated intrathrower reliability for arm speed and shoulder rotation [13]. Improvements in this technology may enable a cost-effective method to monitor throwing athletes’ form and cumulative elbow stress to minimize the risk of injury.

Another important clinical realm where wearable sensors demonstrate potential is the monitoring of rehabilitation progress following knee surgery. For example, post-operative total knee arthroplasty (TKA) or anterior cruciate ligament reconstruction patients whose ROM is not progressing may be identified early to help prevent the need for manipulation under anesthesia or an arthroscopic lysis of adhesions. A systematic review of 14 investigations - 11 of which included patients who underwent TKA or unicompartmental knee arthroplasty (UKA) - concluded that remotely monitored rehabilitation exhibited similar outcomes as a conventional post-operative physical therapy program [14]. However, it is important to note the heterogeneity of the included studies; only five used dedicated knee sensors, while the other nine utilized general physical activity sensors. Additionally, many of the dependent variables were patient-reported outcome measures rather than strictly ROM. A different investigation of 34 participants examined the agreement between knee flexion measured by a combination of lateral thigh and lower leg sensors versus a criterion motion capture system [15]. The authors reported that the root mean square error was less than 3° during walking and stair navigation; therefore, they concluded that the wearable sensors accurately measured knee flexion. Although the study was intended to validate the use of wearables to track ROM following TKA, none of the patients had undergone previous surgery. Furthermore, only knee flexion, but not extension, was measured.

The present study has several limitations. First, all participants were healthy and devoid of elbow or knee pathology. Because the purpose of the sleeve is to obviate the need for a goniometer in the clinical setting, the intended target population may possess coronal or sagittal plane deformities, such as patients with osteoarthritis or ligamentous insufficiency, that would likely impact the sleeve’s measurements in its current form [16]. Second, inter-rater and intra-rater reliability were not established for the two examiners recording the goniometer measurements; therefore, it is possible that some inconsistency exists, despite good reliability in the upper extremity [17]. This could have influenced the observed bias and LoA values. Finally, difficulty with precisely positioning the sleeve, primarily in achieving the correct orientation of the sensor and interconnects around the elbow, may have also contributed to the error. Future investigations should explore the utility of these wearables in patients with joint pathology, using robust calculations of agreement, and further comparison calculations including a larger sample size as well as inter-rater and intra-rater reliability.

## Conclusions

The proprietary sleeve investigated in this study demonstrated significant systematic bias relative to the traditional goniometer; therefore, the sleeve needs refinement, and thus, we cannot recommend that it be utilized in the clinical setting at this time. While the device shows some promise, primarily for measuring knee extension, it currently requires substantial refinement before it can obviate the need for the traditional goniometer. Part of this bias may be due to the challenges faced in consistent sleeve placement around the joint. Most importantly, improvements in product development should focus on optimizing the sleeve fit and the reproducible positioning of the strain sensor to address the user-interface challenges present in the current study. Consistent and precise sleeve placement should enhance clinical utility. Further research must demonstrate validity in not only healthy patients but also those with joint pathologies, such as osteoarthritis and ligamentous insufficiency, and include a larger sample size. This study serves as a valuable foundation for this technology, pointing to the possibility that with reduced bias, wireless sleeves may present a new, less cumbersome alternative for measuring joint ROM.
